# The eastern swamp crayfish *Gramastacus lacus* sp. n. (Decapoda, Parastacidae) a new species of freshwater crayfish from coastal New South Wales, Australia

**DOI:** 10.3897/zookeys.398.7544

**Published:** 2014-04-04

**Authors:** Robert B. McCormack

**Affiliations:** 1 Australian Crayfish Project, c/-Australian Aquatic Biological Pty Ltd, P.O. Box 3, Karuah, NSW, 2324 Australia

**Keywords:** *Gramastacus lacus*, Australia, Central Coast, Mid North Coast, Parastacidae, Ramsar wetlands

## Abstract

*Gramastacus lacus* **sp. n.**, is described from coastal lowlands of the Central and Mid North Coast regions of New South Wales, Australia. *Gramastacus lacus* has a restricted distribution in ephemeral habitats, being dependent on regular natural flooding and drying cycles, and burrows for survival during temporary dry cycles. Documented are population distributions in lowland habitats (3–48 m, a.s.l.) from Wamberal Lagoon, north along the coastal strip to Wallis Lake. The species is small, reaching a maximum weight of 7 grams and 21.3 mm OCL, and distinguished by a large male genital papilla, large raised post orbital ridges, laterally compressed carapace and elongated chelae.

## Introduction

**Figure 1.**
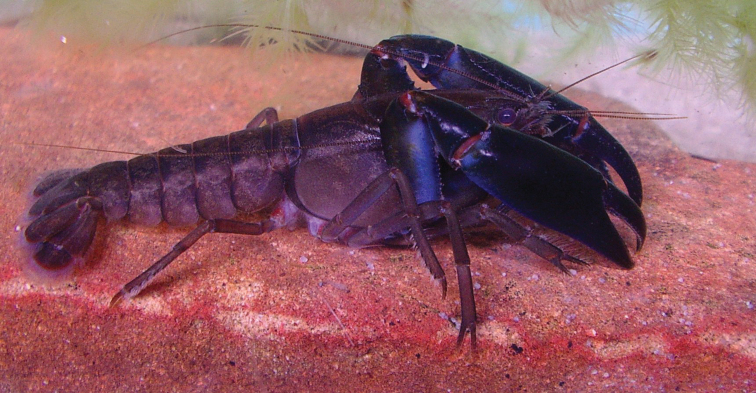
The eastern swamp crayfish *Gramastacus lacus* sp. n.

**Figure 2.**
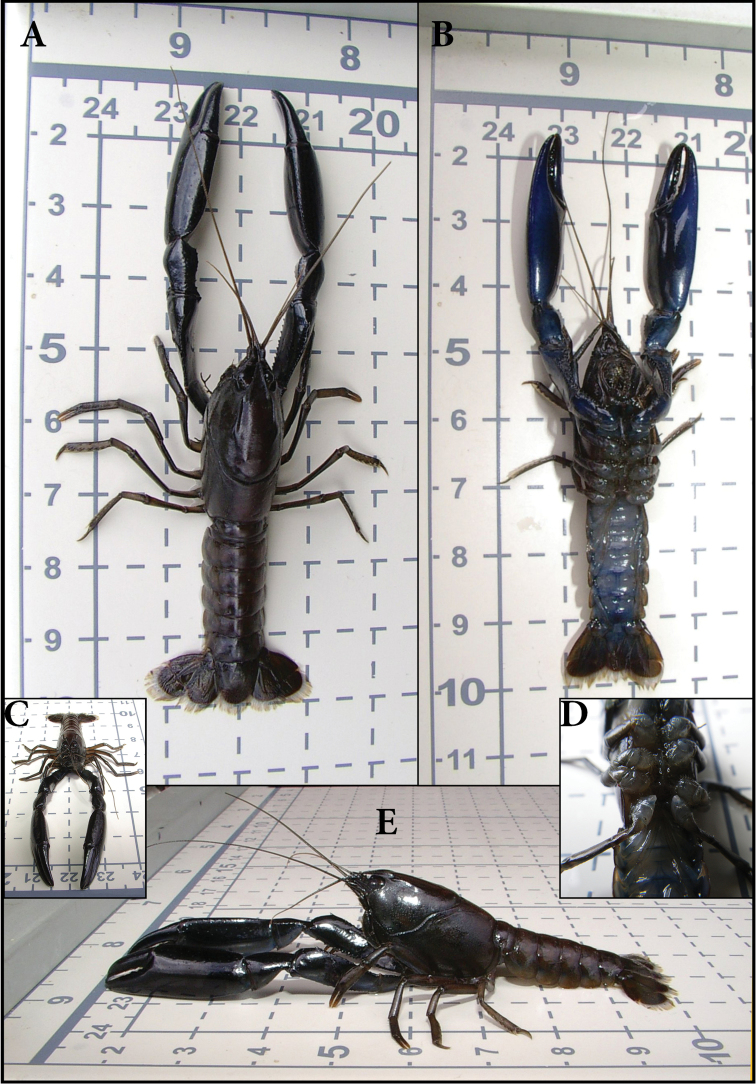
*Gramastacus lacus* sp. n. Holotype male, AM P.89665, 18.63 mm OCL, 5.07 grams. **A** dorsal view **B** ventral view **C** anterior view **D** male genital papilla **E** lateral view. All measurements in centimetres.

**Figure 3.**
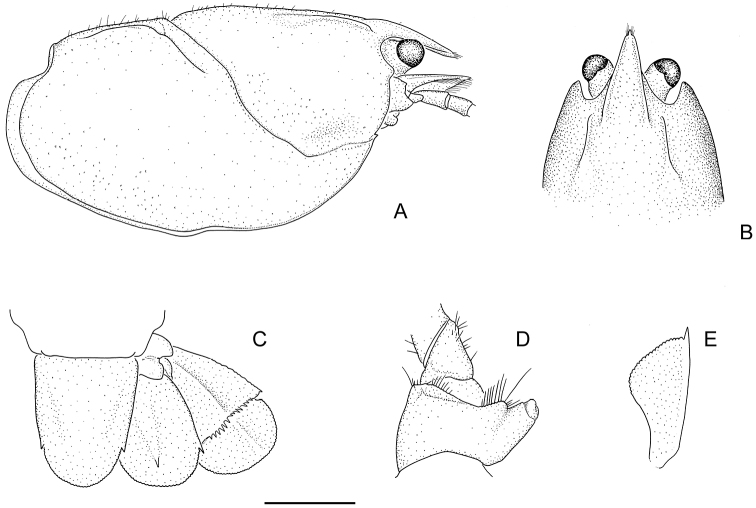
Holotype male: **A** carapace, right lateral view **B** anterior of carapace, dorsal view **C** telson and right uropod (setae omitted) **D** coxa of right pereopod 5 with gonopore, posterior view **E** right scaphocerite (antennal squame), dorsal view (setae omitted). Scale **A–C** = 5mm; **D–E** = 2.5 mm.

*Gramastacus lacus* sp. n., first came to my attention in 1984 when specimens were collected from the Ramsar Wetlands of Myall Lakes National Park for aquaculture trials. Since 2005 the Australian Crayfish Project (ACP) has been surveying eastern Australia to increase the knowledge base of all freshwater crayfish species. As part of this ongoing project, surveys of coastal New South Wales (NSW) over the last eight years have resulted in the discovery of isolated populations of this new *Gramastacus* species (Figures 1–3).

Riek (1972) erected the genus *Gramastacus* comprising two new species, *Gramastacus insolitus* Riek, 1972, and *Gramastacus gracilis* Reik, 1972, from the Grampians area of western Victoria. Zeidler and Adams (1990) revised the genus and concluded that *Gramastacus gracilis* was a junior synonym making the genus monotypic. This new species from coastal NSW, occurring 900 km northeast of *Gramastacus insolitus*, brings the species total back to two.

Schultz et al. (2007) included specimens of the then undescribed New South Wales *Gramastacus* in their study and concluded from a molecular perspective, that these specimens are clearly *Gramastacus*, despite their wide geographic separation from the only currently recognized species in this genus. In this paper I present a formal description of the new species, together with biological and distribution information.

## Methods

Crayfish were collected using a variety of methods to suit the conditions at each survey site. Opera House traps (630 mm × 470 mm × 180 mm, 90 mm steel ring entrance hole) and box traps (430 mm × 260 mm × 260 mm, 50 mm steel ring entrance hole) were utilized, all baited with fresh fish. Scoop rakes and scoop nets were used to sample suitable sites with collection by hand used at many sites. Such collection included lifting structures like rocks and logs and excavating burrows, using hands and with the assistance of spades. Burrows were carefully and slowly excavated allowing the burrow and any branches to be followed. Careful burrow excavation provides information on species habitat requirements and burrow system structures.

In an effort to better understand the biology of freshwater crayfish throughout their ranges and over time, voucher material for further study was retained where appropriate (to confirm the distribution and occurrence of a species at a certain place at a certain time). All retained specimens were placed in plastic transport containers with a small amount of water and returned alive to the laboratory. Specimens were photographed and examined in the laboratory under dissecting microscopes; measured with digital calipers; and weighed to nearest gram. Specimens were euthanized by freezing for at least 24 hours and subsequently stored in clear, labelled specimen jars containing originally 70% ethanol (and now 100% ethanol to better preserve tissue integrity for our genetics collaborations with James Cook University and the Australian Museum). Additionally, tissue samples from live animals were retained in cell lysis buffer from selected specimens for subsequent DNA analysis, as part of the broader ACP via our Carnegie Museum of Natural History genetics program.

Type material is deposited in the Australian Museum, Sydney (AM). Other voucher specimens are lodged in the ACP collection, the Australian Museum and the Carnegie Museum of Natural History (CMNH). Additionally, results of surveys are included in the Atlas of NSW Wildlife database.

At each site, the geographic position co-ordinates and altitude were recorded using a Magellan Explorist 510 and 600 handheld GPS. Notes were taken on exact location details, landforms, aquatic vegetation and stream conditions. Water quality information (flow, pH, temperature, salinity, visibility, DO, conductivity and TDS) was also recorded at selected sites. All specimen measurements are given in millimetres and all weights in grams. Morphological abbreviations and measurements follow Morgan (1986).

### Abbreviations

a.s.l. above sea level

ACP Australian Crayfish Project

AdW abdominal width

AM Australian Museum

ArL areola length; measured along the midline from the cervical groove to dorsal posterior of carapace.

ArW areola width; minimum width of areola.

CD carapace depth; maximum depth at the deepest part, from dorsal carapace to ventral margin above legs.

CL carapace length; measured along the midline from apex of rostrum to the dorsal posterior margin of the carapace.

CMNH Carnegie Museum of Natural History

CW carapace width; taken arbitrarily, maximum width at the widest point.

DactL dactylus length of the first cheliped

LP lateral process

OCL occipital carapace length; measured from the posterior margin of the orbit to the dorsal posterior of carapace (Morgan 1986).

PropD first cheliped propodus depth; greatest thickness, measured between dorsal and ventral palm surfaces.

PropL first cheliped propodus length; measured from propodal base to apex propodal finger.

PropW first cheliped propodus width; greatest height, measured between proximal and distal edges of mesial margin of propodus.

RL rostral length

RW rostral width

Spread TAP minus TAA

SqL antennal squame length.

TAA teeth anterior to the margin of zygocardiac ossicle ear

TAL total abdomen length

TAP teeth anterior to posterior margin of zygocardiac ossicle ear

TEL tailfan; measured from posterior margin of abdominal somite 6 to the tip of the telson

TL total length; measured from the apex of the rostrum to the tip of the tailfan

## Taxonomy

### Gramastacus lacus sp. n.

http://zoobank.org/FCA312BD-AB04-49B3-9177-C50C13D93F78

http://species-id.net/wiki/Gramastacus_lacus

Figures 1 – 3

#### Material examined.

HOLOTYPE: AM P.89665, male, 18.63 mm OCL, 5.07 grams, Boomeri Swamp behind Boomeri camp ground, off Old Gibber Road, Myall Lakes National Park, 32.50910°S, 152.32143°E, altitude 12 m, 21 September 2012, R.B. McCormack.

ALLOTYPE: AM P.89663, berried female, 16.86 mm OCL, 3.17 grams, type locality, 21 September 2012, R.B. McCormack.

PARATYPES: AM P.89664, male, 17.62 mm OCL, 4.55 grams, type locality, 21 September 2012, R.B. McCormack. AM P.89666, berried female, 14.23 mm OCL, 2.06 grams, type locality, 21 September 2012, R.B. McCormack. AM P.89660, male, 20.22 mm OCL, 7 grams, tributary of Boolambayte Creek crossing Violet Hill Rd, Boolambatye, 32.41893°S, 152.28788°E, altitude 21 m, 19 August 2009, R.B. McCormack and S. Pavescki. AM P.89661, male, 16.76 mm, OCL, 4 grams, tributary Pourmalong Creek, under power lines, off Wyee Road, Wyee, 33.12770°S, 151.47438°E, altitude 36 m, 23 August 2009, R.B. McCormack. AM P.89662, male, 17.36 mm OCL, 4 grams, wetland swamp, Wamberal, 33.40927°S, 151.45890°E, altitude 7 m, 10 June 2010, R.B. McCormack.

#### Other material examined.

CMNH 37973.83, female, 14.47 mm OCL, Myall Lakes, May 2008, R.B. McCormack and K. Dawkins. CMNH 37973.84, male, 17.75 mm OCL, Myall Lakes, May 2008, R.B. McCormack and K. Dawkins. AM P.78514, Smiths Lake, May 1970, J. Paxton. ACP 179, male, 19.36 mm OCL, Myall Lakes, August 2005, R.B. McCormack. ACP 180, berried female, 15.27 mm OCL, Myall Lakes, August 2005, R.B. McCormack. ACP 181, male, 16.79 mm OCL, Myall Lakes, August 2005, R.B. McCormack. ACP 806, male, 2 g, 13.96 mm OCL, Myall Lakes, August 2007, R.B. McCormack. ACP 1147, male, 17.7 mm OCL, Budgewoi Lake, April 2008, R.B. McCormack. ACP 1148, male, 17.85 mm OCL, Myall Lakes, April 2008, R.B. McCormack. ACP 1169, male, 2 g, 14.65 mm OCL, Myall Lakes, May 2008, R.B. McCormack, K. Dawkins. ACP 1170, female, 2 g, 16.54 mm OCL, Myall Lakes, May 2008, R.B. McCormack, K. Dawkins. ACP 1171, male, 2 g, 14.65 mm OCL, Myall Lakes, May 2008, R.B. McCormack, K. Dawkins. ACP 1172, male, 3 g, 15.7 mm OCL, Myall Lakes, May 2008, R.B. McCormack, K. Dawkins. ACP 1591, male, 14.06 mm OCL, Lake Macquarie, November 2008, R.B. McCormack. ACP 1598, female, 9.17 mm OCL, Lake Macquarie, November 2008, R.B. McCormack. ACP 1628, male, 13.06 mm OCL, Lake Macquarie, November 2008, R.B. McCormack. ACP 1630, male, 12.13 mm OCL, Lake Macquarie, November 2008, R.B. McCormack. ACP 1651, male, 14.7 mm OCL, Lake Macquarie, December 2008, R.B. McCormack, S. Pacevski, J. Moylan, T.A. Moylan. ACP 1652, male, 13.38 mm OCL, Lake Macquarie, December 2008, R.B. McCormack, S. Pacevski, J. Moylan, T.A. Moylan. ACP 2286, female, 3 g, 16.7 mm OCL, Myall Lakes, August 2009, R.B. McCormack, S. Pacevski. ACP 2300, berried female, 3 g, 16.74 mm OCL, Lake Macquarie, August 2009, R.B. McCormack. ACP 2306, female, 3 g, 15.88 mm OCL, Lake Macquarie, August 2009, R.B. McCormack. ACP 2307, berried female, 4 g, 17.56 mm OCL, Lake Macquarie, August 2009, R.B. McCormack. ACP 2309, berried female, 2 g, 15.21 mm OCL, Lake Macquarie, August 2009, R.B. McCormack. ACP 2313, female, 7 g, 21.32 mm OCL, Myall Lakes, August 2009, R.B. McCormack. ACP 2318, berried female, 1 g, 12.3 mm OCL, Myall Lakes, September 2009, R.B. McCormack. ACP 2349, male, 11.99 mm OCL, Smiths Lake, September 2009, R.B. McCormack. ACP 2354, female, 9.2 mm OCL, Wallis Lake, September 2009, R.B. McCormack. ACP 2363, male, 11.79 mm OCL, Wallis Lake, September 2009, R.B. McCormack. ACP 2368, female, 9.08 mm OCL, Wallis Lake, September 2009, R.B. McCormack. ACP 2369, female, 11.27 mm OCL, Wallis Lake, September 2009, R.B. McCormack. ACP 2376, female, 11.72 mm OCL, Wallis Lake, September 2009, R.B. McCormack. ACP 2378, berried female, 1 g, 13.4 mm OCL, Myall Lakes, September 2009, R.B. McCormack. ACP 2387, berried female, 2 g, 13.08 mm OCL, Myall Lakes, September 2009, R.B. McCormack. ACP 2403 berried female, 2 g, 14.1 mm OCL, Myall Lakes, September 2009, R.B. McCormack. ACP 2411, male, 2 g, 15.1 mm OCL, Budgewoi Lake, September 2009, R.B. McCormack. ACP 2422, berried female, 1.5 g, 13.68 mm OCL, Tuggerah Lake, September 2009, R.B. McCormack. ACP 2532, male, 13.53 mm OCL, Port Stephens, December 2009, R.B. McCormack. ACP 2925, mix x 2, Wamberal Lagoon, May 2010, R.B. McCormack. ACP 2926, male, 4 g, 15.57 mm OCL, Wamberal Lagoon, May 2010, R.B. McCormack. ACP 2928, male, 3 g, 14.35 mm OCL, Wamberal Lagoon, May 2010, R.B. McCormack. ACP 4059, male, 3.65 g, 17.67 mm OCL, Myall Lakes, July 2012, R.B. McCormack. ACP 4063, female, 2.37 g, 16.46 mm OCL, Myall Lakes, July 2012, R.B. McCormack. ACP 4072, male, 2.99 g, 16.13 mm OCL, Myall Lakes, July 2012, R.B. McCormack. ACP 4074, female, 2.84 g, 16.52 mm OCL, Myall Lakes, July 2012, R.B. McCormack. ACP 4079, male, 4.26 g, 17.7 mm OCL, Myall Lakes, July 2012, R.B. McCormack. ACP 4100, female, 1.21 g, 12.91 mm OCL, Myall Lakes, September 2012, R.B. McCormack. ACP 4101, male, 1.97 g, 14.48 mm OCL, Myall Lakes, September 2012, R.B. McCormack. ACP 4102, male, 2.56 g, 14.84 mm OCL, Myall Lakes, September 2012, R.B. McCormack. ACP 4109, male, 0.83 g, 10.81 mm OCL, Myall Lakes, September 2012, R.B. McCormack. ACP 4114, male, 0.61 g, 9.77 mm OCL, Myall Lakes, September 2012, R.B. McCormack. ACP 4116, female, 1.11 g, 12.10 mm OCL, Myall Lakes, September 2012, R.B. McCormack. ACP 4121, female, 1.94 g, 14.66 mm OCL, Myall Lakes, September 2012, R.B. McCormack. ACP 4130, male, 1.39 g, 12.24 mm OCL, Myall Lakes, September 2012, R.B. McCormack.

#### Comparative material examined.

ACP 899, *Gramastacus insolitus*, male, 10 mm OCL, Moora Moora, Grampians, Victoria. ACP 927, *Engaeus laevis*, male, 15.81 mm OCL, Thurra River, Croajingalong, Victoria. ACP 2884, *Engaeus lyelli*, male, 22.54 mm OCL, Eildon, Victoria.

#### Diagnosis.

Rostrum long, narrow, reaching midlength or end of 3rd antennal segment, with spine at apex. Rostral carinae conspicuously raised and sharp, extending well back onto carapace just inside postorbital carinae. Carapace laterally compressed, deep, narrow. Antennal flagellum twice OCL. Antennal squame very long reaching end of 3rd antennal segment or beyond, widest at midlength, usually half as wide as long. Antennal basipodite spine variable, small to very large and sharp; coxopodite antennal spine absent. Interantennal spine wide, margins smooth and raised, with blunt to sharp spine. Areola very broad, width 0.5–0.7 times length, narrowest at centre. Telson U-shaped, margins gently converging to caudolateral corners, each with small sharp spine. Uropod outer ramus with 2 medium marginal outer spines with tuft of setae between. First chelae smooth and distinctly elongated. Males with large genital papilla.

#### Description.

**Size.** Maximum OCL 21.32 mm, 7 gram. Maximum size animals extremely rare, mean weight of large adults 4-5 gram.

**Rostrum.** Long, narrow, reaching midlength or end of 3rd antennal segment, approximately 0.3 × OCL. Rostral apex variable, ranging from very small to a large sharp conical acumen spine; apex upturned at 45–60° angle. Rostral carinae conspicuously raised and sharp, extending well back onto carapace, reaching beyond anterior end of postorbital carina; intracarinate region flat or slightly recessed, setose along mesial base of carinae with tuft of setae at apex of rostrum, carinae terminating at base of acumen in blunt process to large sharp conical rostral marginal spine upturned at 30° angle. OCL/CL 0.78–0.86. RW/OCL 0.09–0.14. RL/OCL 0.25–0.39.

**Cephalon.** Postorbital carinae conspicuously raised and prominent, anteriorly extremely variable from blunt to large sharp conical spine. Carinal length similar to rostral length. Suborbital spine small to medium. Antennal squame long, reaching to end of 3rd antennal segment or beyond, lateral margin slightly convex terminating in long, sharp conical spine, inflated at midlength. Cephalic setation variable, from slight to medium with scattered long bristle setae, densest towards lateral cephalic edge. SqL/OCL 0.18–0.32.

**Thorax.** Cervical groove deeply impressed, U-shaped. Postcervical groove lightly impressed, well separated from cervical groove, with postcervical groove diverging laterally; postcervical groove not continuous, vaguely extending just past the inner side of branchiocardiac groove. Branchiocardiac groove merging gradually with postcervical groove, with a distinct, short, transverse groove at posterior end of branchiocardiac groove. Areola very broad, 0.5-0.7 wide as long, narrowest towards centre. Carapace laterally compressed, deep, narrow, depth 0.67 × OCL, width 0.55 OCL. ArL/OCL 0.29–0.39. ArW/OCL 0.19–0.25. CW/OCL 0.51–0.59. CD/OCL 0.65–0.73.

**Abdomen.** Smooth, unarmed, with scattered long bristle setae. Some morphological differences between sexes (see Sexes). TAL average 1.66 × OCL. TAL/OCL 1.5-1.87. AdW/OCL 0.48–0.61 (average 0.54). TL/OCL 2.51-3.13 (average 2.84).

**Tailfan.** Telson U-shaped with sides gently converging to caudolateral corners, each with 1 small sharp standard spine. Outer uropod with 2 small standard marginal outer spines with tuft of setae between, 1 medium spine on longitudinal median carina on suture, longitudinal carina extends beyond suture, suture straight, 5–11 extra dorsolateral spines (outer) and 5–9 extra dorsolateral spines (inner) stopping just past half way. Outer uropod extends well past inner uropod and inner uropod extends just past caudal margin of telson. Inner uropod with medium standard spine at caudolateral corner, small upturned spine at base of longitudinal median carina (two specimens with 2 spines on one side). Tailfan with medium scattered long bristle setae. Coxopodite of the outer uropod terminating in rounded edge to medium conical spine, coxopodite of inner uropod terminating in small to medium conical spine. TEL/OCL; females 0.41–0.47 (average 0.45), males 0.38–0.47 (average 0.42).

**Thoracic sternal keel.** Sternal keel commencing as raised ridge posterior of LP1, thin sharp and recessed between LP1 and LP2. Rising at LP2 with slight dip between LP2 and LP3 with a small crest at LP3 and continuing straight as raised sharp ridge to LP4. LP4 with distinctive huge oval pores posterolaterally. Bullar lobes absent.

**Gastric mill.** TAP count 2.5–3.5. TAA 0.5–1.5. Spread 1–2. Urocardic ridges 2.

**Antennae.** Basipodite antennal spine small to very large and sharp. Coxopodite antennal spine absent. Interantennal spine wide, smooth raised margins, with blunt to pointed apex. Antennal flagellum twice OCL reaching posteriorly to 5th or 6th abdominal somites.

**Third maxilliped.** Third maxilliped with large raised spiniform process at mesioventral corner of coxopodite; ischium with sparse covering of short to long setae on ventrolateral surface, with carinate lateral edge and tuberculate laterodistal corner; exopodite multiarticulate and very long, 1.33–2.08 as long as ischium.

**First cheliped.** Distinctively elongated, proportionally larger in mature males than in reproductive females. General increase in setation along propodal and dactylar cutting edges in populations from the north to the south.

*Propodus*. Lightly punctated with mesial propodal margin starting as small rolled ridge at carpal articulation, progressing to small rounded mesial propodal spines scattered in twin lines giving a shadow effect to dactylar articulation. Cluster of small protuberances, some approaching small spines at perpendicular groove behind dactylar articulation. Dorsal and ventral propodal palm surfaces smooth. Shallow ventromesial and ventrolateral groves extending from apex along finger. Small granulations on lateral propodal edge usually (only distal 2/3 towards apex) but not on propodal finger. Light to medium setation along cutting edge with 2 prominent teeth.

Males (mean): PropL/OCL (1.07) 0.82-1.35, PropW/PropL (0.37) 0.33–0.44, PropD/PropL (0.255) 0.22–0.28.

Females (mean): PropL/OCL (0.87) 0.7–1.06, PropW/PropL (0.375) 0.30–42, PropD/PropL (0.245) 0.19–0.29.

*Dactylus*. Smooth, lightly punctated, with line of medium to large granulations along mesial edge increasing in size from dactylar articulation to apex between shallow dorsomesial and dorsolateral groves extending back from apex, fading out at approximately 40% dactylar length. One prominent tooth on cutting edge. Light to medium setation along cutting edges, more extensive in southern specimens. DactL/PropL: males (0.475) 0.41–0.54, females (0.51) 0.45–0.57.

*Carpus*. Smooth, with line of 6-9 small spines in irregular row along mesial edge, terminating in large sharp spine at articulation. Cluster of small protuberances in irregular line along ventromesial surface, several rounded, spiniform at articulation. Larger lateral carpal spine at articulation with group of smaller rounded spines along articulation edge. Setation absent.

*Merus*. Medium sharp outer distolateral meral spine, prominent dorsal meral spine. Setation absent.

#### Coloration.

Colour varying with population. First chelipeds very dark, black to black-blue with bright blue highlights along propodal, carpal and meral lateral edges, light blue tint ventrally with articulations dull to bright red. Cephalon dark black-brown dorsally, lightening laterally, many with blue highlight on lateral surface. Thorax and abdomen light brown, green, tan or steel blue, usually with small, light cream or red speckles. Body clear to cream ventrally. Juveniles (Wallis Lake) light blue.

#### Sexes.

No intersexed individuals were recorded, all being clearly either male or female. The male genital papilla is large, being a distinguishing character (Riek 1972) from other crayfish species in the region. They are opportunistic breeders, starting in early August being reliant on water availability in their ephemeral habitats, only breeding when flooded. Females mature at approximately 12 mm OCL and are very fecund for their size: ACP 2318, 12.3 mm OCL, September 2009, Myall Lakes, 44 eggs; ACP 2387, 13.08 mm OCL, September 2009, Myall Lakes, 29 eggs; ACP 2309, 15 mm OCL, August 2009, Lake Macquarie, 82 eggs; ACP 180, 15.93 mm OCL, August 2005, Myall Lakes, 79 eggs; ACP 2300, 16.74 mm OCL, August 2009, Lake Macquarie, 100 eggs; ACP 2307, 17.56 mm OCL, August 2009, Lake Macquarie, 143 eggs. Eggs are dark olive green colour (ACP 2318), to deep dark purple (ACP 2384). Berried females have previously been captured from water after rain events in August, September and October with approximately 30–150 eggs. Egg incubation is swift with a 6–8 week period between eggs laid and juveniles released. Females breeding in early September released juveniles in late October.

*Gramastacus lacus* shows sexual dimorphism. Reproductively active females typically have broader abdomens with an abdominal anterolateral flap on the pleuron of abdominal somite 2 and have a smaller/narrower major cheliped then males. Reproductively active females are recorded between 12 and 18 mm OCL, but more research is needed (berried females over 18 mm OCL have not yet been recorded).

#### Etymology.

The Latin epithet*lacus* alludes to the coastal lake catchments that provide suitable habitat. Common name; previously referred to as “the lake yabby” (McCormack 2008), however, I recommend “the eastern swamp crayfish” as more appropriate, in parallel to the only other recognized *Gramastacus* species, *Gramastacus insolitus*, commonly known as “the western swamp crayfish” (Action Statement 2004).

## Discussion

**Figure 4.**
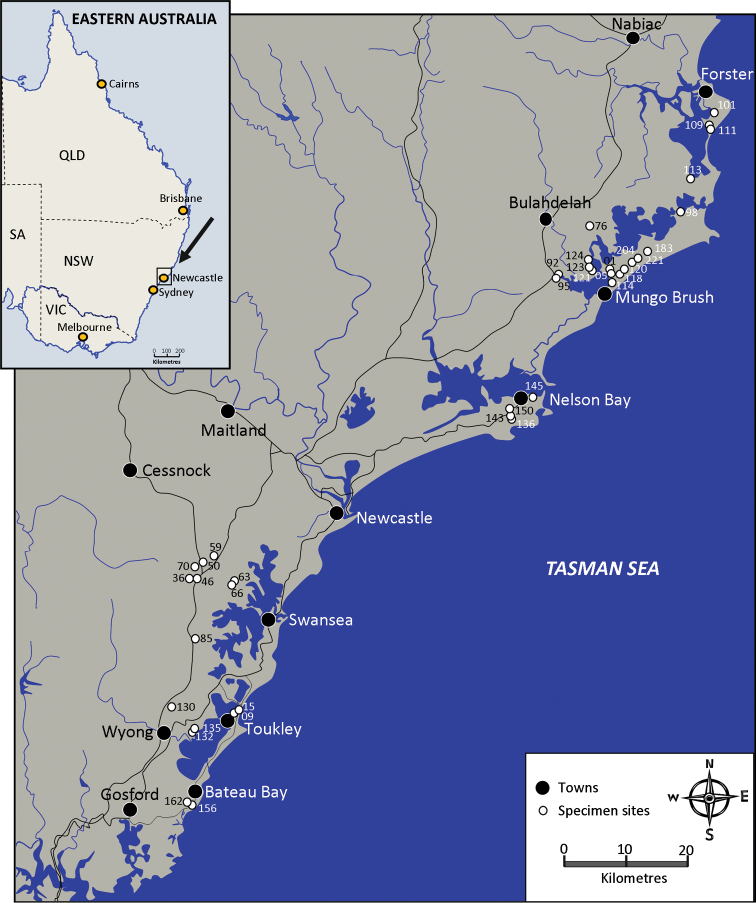
Distribution map. Records of population sites.

*Gramastacus lacus* is a small freshwater crayfish inhabiting lowland coastal environments between 3 and 48 m a.s.l. To date, it is known to occur from Wallis Lake in the north to Wamberal Lagoon in the south — a straight line distance of 165 km (Figure 4, Supplementary material 1). Further south, suitable habitat occurs around Avoca and Cockrone Lagoons (33.4917°S, 151.4201°E) but preliminary surveys have not yet identified the species presence. Further north, past Forster (32.0697°S, 152.5168°E), suitable habitat is also present but the species remains unrecorded. Future field surveys may increase the known species distribution.

Our research indicates that *Gramastacus lacus* has specific habitat requirements, preferring ephemeral habitats (smaller creeks, swamps, wet areas and stump holes) that offer conditions that enable their survival and presumably limit threats from other predators, such as eels. *Gramastacus lacus* populations were only found in ephemeral areas that reliably flood and then dry. Habitats in the same area that consist of permanent water invariably contain large numbers of freshwater eels (*Anguilla reinhardtii*) and native fish gudgeons (family Eleotridae) but not *Gramastacus lacus*. Many ephemeral swamps and creeks can revert from dry beds to water bodies several metres deep when flooded. Eels and fish may penetrate into and temporarily visit some habitat areas but *Gramastacus lacus* takes refuge in the periphery, protected within the thick reeds and flooded grasses along the shallow edges, well away from the deeper water.

Larger swamps, such as Boomeri Swamp, the type locality in the Ramsar Wetlands of the Myall Lakes National Park, will dry out completely and then flood with up to 1.5 m of water. Deep water over 1 m is usually devoid of *Gramastacus* except occasional moulted individuals. The only *Gramastacus* observed in >1 m of water are soft moulted animals, presumably they are seeking the deeper water away from the high density populations in the shallows to avoid cannibalism. We have not captured eels or fish from this swamp but giant water bugs (*Lethocerus insulanus*), turtles (*Chelodina longicollis*), lizards (*Physignathus lesueurii*) and birds all prey in deeper water making it a more dangerous habitat for the *Gramastacus* crayfish.

No quantitative data are available on abundance of *Gramastacus lacus*. From our observations and sampling, however, I estimate that at suitable sites they were abundant with densities up to 35/m^2^ commonly in shallow heavily reeded areas within protected National Parks like the Myall Lakes. In altered habitats and areas impacted by human activity, the population numbers of *Gramastacus lacus* were low, at 1/10 m^2^ around Budgewoi Lake.

*Gramastacus insolitus*, unlike *Gramastacus lacus*, is a very small non-burrowing freshwater crayfish, being commensal with larger crayfish species, using their burrows to survive the seasonal drying of their habitat (Johnston and Robson 2009). *Gramastacus lacus* differs from its congener by being a robust, strong, burrowing species relying on its own burrow for extended survival during drying events. Burrows are relatively simple, with a single entrance and rounded cross-section, generally descending at a steep angle. Depth varies with soil types, but most have a single corridor, penetrating 450 to 750 mm, with older burrows having a small rounded base chamber. Deepest burrows observed extend down about one metre and generally reach the water table. The preferred lowland coastal areas generally have shallow water tables. Burrows are completely round and colloquially referred to as “bore holes”; they may be capped with mud but are generally open.

Only one adult crayfish per burrow was observed. On several occasions (at Myall and Wyee), however, one small juvenile crayfish was found with the adult. Unlike, *Gramastacus insolitus*, *Gramastacus lacus*, does not utilise the burrows of *Cherax* species, and generally other crayfish species are not found within the main habitat areas. In the northern distribution of *Gramastacus lacus*, the species was found in sympatry with *Cherax setosus*, occasionally with *Euastacus reductus* at the limits of its distribution. Typically in small intermittent streams, only *Gramastacus lacus* is found downstream in smaller/shallower burrows where the water tables are shallow, and only *Cherax setosus* is found upstream in much larger deeper burrows with a 20 m intermix zone between where both occur within burrows less than 200 mm apart. Unfortunately, translocated *Cherax destructor* exist amongst the Wamberal Lagoon population of *Gramastacus lacus* and I have grave fears for its future, with the outbreak of *Cherax destructor* being an ongoing significant threat to that population of native *Gramastacus* (McCormack in press).

*Gramastacus lacus* possesses large elongated chelae that are used quite effectively in water to defend themselves and ward off attack from other crayfish, small fish or macroinvertebrates. When submerged they will raise their open claws and wave them rapidly about in defence. However, they are seemingly rarely used for defence out of water. Crayfish are easily handled and will rarely nip when handled. Chelae are long and ungainly out of water, possibly being difficult to raise and hold extended. Individuals tend to rapidly lower their claws and retreat, preferring to avoid confrontation. In backwards retreat, the crayfish will drag the claws or use them in a series of claw over claw pushing motions to help them move more rapidly. If the crayfish moves forward, then it has two types of movement. Firstly, it will individually, alternatingly raise a claw, extend and lower it whilst moving forward till it reaches the claw tips then lift it up and extend it again. This coordinated claw over claw movement is usually only used at the start of the movement then the crayfish generally changes to a more unusual movement that may be easier or more efficient. Secondly, there is a unique forward movement via a series of rhythmic plunges. The crayfish raises the cephalothorax and both claws up with its legs and then moves forward overbalancing and plunges down and forward then repeats the movement. This up and forward movement is unusual, but the crayfish easily moves up, forward and down without “missing a beat”.

Unlike many Australian freshwater crayfish, *Gramastacus lacus* is not known to be subject to temnocephalan infestation, with no records from any sampled populations. Notably, *Cherax destructor*, *Cherax setosus* and *Euastacus reductus* captured together with *Gramastacus lacus* have had healthy populations of temnocephalans. I feel that this is a significant facet of biological information that needs further research. Also noteworthy was one specimen ACP 4122 (in the vicinity of the type locality) with two eggs from an unidentified species attached to the lateral posterior of the carapace.

Further research into the genetics of each catchment population should be a priority. Other than minor setal variation on the first chelipeds, no consistent morphological differences could be identified between populations even though each population seems geographically separated, and some by large marine water barriers, such as the Hunter River and Port Stephens. The degree of genetic connectivity between populations is not yet known. I suspect that populations have been isolated through habitat fragmentation and may be highly divergent and genetically distinct, containing unique haplotypes. If so, these could represent different evolutionary significant units (ESUs) or conservation management units (MUs) that might require individual conservation attention, especially because an important goal of species conservation is to preserve genetic diversity.

As the species occurs along the coastal strip in some of the fastest developing areas of Australia, further field surveying to identify isolated populations should also be a priority, with an assessment of the conservation status of each population as currently nearly all are potentially endangered.

## Supplementary Material

XML Treatment for Gramastacus lacus

## Supplementary material

Supplementary material 1 *Gramastacus lacus* specimen data. doi: 10.3897/zookeys.398.7544.app1 Data type: Specimen data. Explanation note: Specimen record with locality details for known populations of the eastern swamp crayfish *Gramastacus lacus* sp. n., in coastal New South Wales. This dataset is made available under the Open Database License (http://opendatacommons.org/licenses/odbl/1.0/). The Open Database License (ODbL) is a license agreement intended to allow users to freely share, modify, and use this Dataset while maintaining this same freedom for others, provided that the original source and author(s) are credited.Robert B. McCormack
